# A Wholesome Bed

**Published:** 1874-12

**Authors:** 


### A WHOLESOME BED.

A wholesome bed is at last possible. It is safe to say, however, that the majority of all beds in use in the best houses are unclean, and in a good state to induce disease. This will seem strange to our readers, but they will presently see the philosophy of the remark.

By far the largest portion of the beds in use among us are made in whole or in part from some animal substance, chiefly feathers or curled hair. These are naturally and almost uniformly wanting in cleanliness. They impart their own peculiar animal odor to the air about them, and give off an offensive oil to whatever they come in contact with. Besides, the usual manner of gathering and preparing these unwholesome articles, added to the deceptions practiced by the incorporation of substances to cheapen them, renders our beds not unfrequently things to be avoided rather than to be sought with assurances of calm and sweet repose. It stands to reason that vegetable substances would prove more healthful and infinitely more agreeable than animal fibres for this purpose, provided the essential condition of permanent elasticity could be secured. The tendency of vegetable fibres is to pack and consolidate, a characteristic which is perhaps less apparent in feathers and curled hair. For many years cotton was employed, and was greatly admired for its sweetness, but its tendency to "mat," pack, and become uneven and depressed in spots, after continued use, rendered it unpopular.

But some years ago an ingenious Frenchman discovered a method of forming cotton into a light and very elastic felt, and from that moment it was found possible to make pillows, mattresses and cushions, possessing quite as much elasticity as the best hair, and with no tendency to become solid after much wear. To accomplish this a fine quality of cotton was required, which, after being perfectly cleaned, was subjected to intense heat, and afterwards treated with a chemical solution, which imparted to it the felting or interlacing power. It was then belted into the light and airy sheets of which we have spoken, and these sheets were piled one upon another until the desired thickness was reached. The structure was then covered with a tick, if a pillow or bed, and upholstered appropriately, if designed for a cushion of any kind.

This business was entered upon just before the late war, but was abandoned as the cost of raw cotton increased. Some who bought the goods when cotton was low were tempted to sell as cotton went up, and have since regretted it, for they have never been able to buy as good a bed at any price. Other early purchasers were not drawn into selling by the high prices, and now declare that their beds are absolutely perfect after fourteen or fifteen years of active and constant use.

These Elastic Felt Mattresses are very appropriately named. We first heard of them a few weeks ago in New Brunswick, New Jersey, and here they had been in use for a number of years, and their excellence and elasticity was very earnestly vouched for by the owner. We obtained the name of the dealer who sold them, and on visiting him we gathered a good many interesting facts concerning the goods. Mr. Brown, of 56 Montgomery Street, Jersey City, informed us that Mrs. Job Cleveland, recently deceased, was wholly unable to rest comfortably in bed during her long illness until the Elastic Felt Mattress was substituted for the very best hair mattress which it was in his power to make. He further stated that the wife of Major Z. K. Pangborn, editor of the Jersey City *Daily Journal*—a confirmed invalid—has experienced the greatest relief from the Elastic Felt Mattress.

Other cases of a like nature were related by Mr. Brown, who was very enthusiastic in his endorsement of the article. We afterwards visited Mr. H. D. Ostermoor, 75 Pearl Street, New York, who is the sole manufacturer in the United States of this patent elastic substance. He showed us cushions which had been a number of years in use, and which had not become flattened by long and severe usage. We examined a mattress which had been in constant wear for eight years, and we could not discover that it was less elastic and plump than those which had never been on a bed.

We were shown a large number of original letters from hospitals, hotel proprietors, railroad companies, car builders, clergymen and church committees, all of whom had tested the Elastic Felt as bedding, or in the form of cushions, and the testimony was strong and unusual in its favor.

A point of importance is that it costs scarcely half as much as good curled hair; while the manufacturer guarantees that a cushion, mattress, pillow, bolster, or any other article filled with his Patent Elastic Felt, will remain permanently sweet, elastic and soft, without being made, even in an ordinary lifetime. This is, of course, worthy of consideration; but we must deem its sanitary advantage vastly more important than its cost. It is wholly free from moth and other insect life, and is infinitely more agreeable to think and talk about than the unclean hair which is obtained from South American horses, and shipped to market with all its natural impurities adhering to it. It is abominable to bring such unclean substances in close contact with our bodies, and it is fair to presume that many diseases have been engendered by the proximity. To affirm that the body is sufficiently protected by the ticking and the sheet is an assumption not warranted by the fact. These fabrics soon become saturated by the exhalations from the skin, and act as ready conductors of whatever impurity the hair may hold. We can adduce about as many arguments against feathers as against hair, and we entertain a strong hope that both may be speedily superseded by the pure white and wholesome cotton felt.

